# Plasma lipid profiling of different types of hepatic fibrosis induced by carbon tetrachloride and lomustine in rats

**DOI:** 10.1186/s12944-016-0244-1

**Published:** 2016-04-12

**Authors:** Masaki Ishikawa, Kosuke Saito, Hiroshi Yamada, Noriyuki Nakatsu, Keiko Maekawa, Yoshiro Saito

**Affiliations:** Division of Medical Safety Science, National Institute of Health Sciences, 1-18-1 Kamiyoga, Setagaya, Tokyo, 158-8501 Japan; Toxicogenomics Informatics Project, National Institutes of Biomedical Innovation, Health and Nutrition, 7-6-8 Saitoazagi, Ibaraki, Osaka, 567-0085 Japan

**Keywords:** Hepatic fibrosis, LC/MS, lipidomics, Plasma lipid profiling, Hepatotoxicity

## Abstract

**Background:**

Plasma lipid profiling has emerged as a useful tool for understanding the pathophysiology of hepatic injury and disease. Hepatic fibrosis results from chronic, progressive damage to the liver and can lead, in turn, to more serious conditions such as hepatic cirrhosis and hepatocellular carcinoma. Thus, the present study aimed to investigate the plasma lipid profiles of two types of hepatic fibrosis in order to aid the understanding of the pathophysiology of hepatic fibrosis.

**Methods:**

A liquid chromatography and mass spectrometry platform was used to reveal and compare the plasma lipid profiles of two types of chemical-induced hepatic fibrosis. Rat models of centrilobular fibrosis and bile duct fibrosis were established via chronic exposure to the known fibrogenic hepatotoxins, carbon tetrachloride (CCl_4_) or lomustine (LS), respectively, over a 28-day period. To delineate the specific alterations in the lipid profiles as a result of the hepatic fibrosis, we also employed non-fibrogenic hepatotoxicants (2-acetamidofluorene, N-nitrosodiethylamine, and ethambutol) as well as 3-day treatment of CCl_4_ and LS, which did not induce fibrosis.

**Results:**

Our assay platform identified 228 lipids in the rat plasma, and the global lipid profile clearly distinguished these models from the control via principal component analysis. In addition, the alteration of the plasma lipid profile caused by CCl_4_ and LS were clearly different. Furthermore, a number of lipids were identified as specific alterations caused by fibrosis induced only by CCl_4_ and LS, respectively. Three lysophosphatidylcholines (LPC[18:3], LPC[20:4], and LPC[22:6]), and three phosphatidylcholines (PC[18:2/20:4], PC[40:8], and PC[20:4/22:6]) are specific circulating lipids, the levels of which were altered by both CCl_4_ and LS treatment; however, their levels were decreased by chronic exposure to CCl_4_ and increased by chronic exposure to LS.

**Conclusions:**

These results suggest that different types of chemical-induced hepatic fibrosis demonstrate clear differences in their plasma lipid profiles. Our study provides insights into the alteration of plasma lipidomic profiles as a result of the fibrosis of different parts of the hepatic lobule, and may help to understand the pathophysiology of different types of hepatic fibrosis.

**Electronic supplementary material:**

The online version of this article (doi:10.1186/s12944-016-0244-1) contains supplementary material, which is available to authorized users.

## Background

Lipids, such as phosphoglycerolipids, sphingolipids, and neutral lipids, are components not only of cellular membranes, but also of blood-circulating lipoproteins. In addition, these lipids play important roles in multiple biological processes, including apoptosis, inflammation, proliferation, and differentiation [1–4]. Aberrations of the tightly regulated lipid homeostasis mechanisms are associated with hepatic injury and disease. Thus, the characterization of the alteration of lipids associated with such diseases and injuries would help in understanding their pathophysiology. It is well known that the synthesis of lipoproteins is one of the key functions of the liver. In addition, a recent study demonstrated that the composition of the plasma lipids is closely correlated with the lipids in the liver [5]. Therefore, plasma lipid profiling could be a useful tool for understanding hepatic pathophysiology. In fact, plasma lipid profiling has already been applied to understanding the pathophysiology, and to identifying biomarkers for hepatocellular carcinoma [6, 7], liver phospholipidosis [8], and non-alcoholic fatty liver disease [9].

Hepatic fibrosis can be defined as the accumulation of extracellular matrix proteins that form excessive connective tissue in the liver in the setting of etiologically diverse hepatic conditions such as alcoholic and nonalcoholic liver disease, viral hepatitis, and disease due to chemicals. In time, this may progress to more serious conditions, such as cirrhosis and hepatocellular carcinoma [10–12]. Thus, understanding the pathophysiology of hepatic fibrosis is important and plasma lipid profiling could be a useful tool for achieving this. In addition, fibrosis may occur in different sections of the classical hepatic lobule, depending on the agent causing the insult. For instance, alcohol-induced fibrosis primarily affects the centrilobular regions [13], whilst infection with the hepatitis C virus primarily affects the portal area [14]. Chronic exposure to the hepatotoxin carbon tetrachloride (CCl_4_) consistently causes fibrosis in the centrilobular regions [15], whilst that induced by lomustine (LS) causes fibrosis in the bile ducts [16]. In the previous literature, the two main functions of lipid homeostasis are also localized in specific areas of the hepatic lobules. Catabolism (fatty acid oxidation and phospholipid degradation) occurs mainly in the periportal area, while anabolism (fatty acid synthesis and fatty acid incorporation into phospholipids and cholesterol esters [ChEs]) is more predominant in the centrilobular area [17, 18]. Thus, the lipid alterations caused by hepatic fibrosis could vary between different regions within the lobule where the insult occurs.

In the present study, we used a rat model to investigate the plasma lipid profiles of two types of hepatic fibrosis. Centrilobular fibrosis and bile duct fibrosis were chemically induced by CCl_4_ and LS, respectively. To analyze the plasma lipid profiles, we employed a recently developed lipidomics platform comprising liquid chromatography and mass spectrometry (LC/MS), which simultaneously determined the levels of a broad spectrum of lipids. In total, we determined and examined 228 lipids (97 phospholipids, 23 sphingolipids, and 108 neutral lipids). The principal component analysis clearly demonstrated differences in the plasma lipid profiles of rats with hepatic fibrosis against those of control rats. In addition, we addressed the specific differences in the lipid alterations of CCl_4_- and LS-induced hepatic fibrosis. We then identified specific plasma lipid molecules associated with different types of chemical-induced hepatic fibrosis.

## Methods

### Animal specimens

The rat plasma samples used in the present study were obtained from the toxicogenomics projects TGP and TGP2 (pathophysiological data were deposited in the Open TG-GATEs database [http://toxico.nibiohn.go.jp/]) [19]. The drug treatments and major histological findings are summarized in Table 1. In brief, 6-week-old male Crl:CD (SD) rats (Charles River Japan, Kanagawa, Japan) were subjected to continuous treatment with the fibrogenic chemicals, CCl_4_ or LS, for either 3 or 28 days (i.e. chronic exposure over 28 days is known to consistently induce hepatic fibrosis). For comparison purposes, either 2-acetamido-fluorene (AAF), N-nitrosodiethylamine (DEN), or ethambutol (ETB) was administered for 28 days as a non-fibrogenic hepatotoxic control. Each drug-treatment group (*n* = 5) had its own corresponding negative control group that was treated with the vehicle only (*n* = 5). At the end of the trial, venous blood and livers were isolated from the rats. Whole blood was subjected to isolate the plasma fraction. The livers were fixed, paraffin-embedded, and subjected to histopathological analyses to evaluate the extent of the hepatic fibrosis and hepatopathy. The use of the animal specimens was approved by the Ethics Review Committee for Animal Experimentation of the National Institute of Biomedical Innovation, Health, and Nutrition (Osaka, Japan) and the National Institute of Health Sciences (Tokyo, Japan).

**Table 1:** Summary of compounds, animals, and histological findings

				Number of animals				Liver histopathology				
Compound name	Abbreviation	Dose (mg · kg^−1^ · day^−1^)	Duration (days)	Control	Chemicals	Administration	Vehicle control	Fibrosis	Cellular infiltration	Necrosis	Proliferation	Ground glass appearance
Carbon tetrachloride	CCL_4_	100	28	5	5	peroral	corn oil	Centrilobular, 5/5 (minimal)	Centrilobular	-	-	-
Lomustine	LS	6	28	5	5	peroral	0.5 w/v% methylcellulose	Bile duct, 1/5 (minimal), 3/5 (mild)	Periportal	Midlobular	Bile duct, Kupffer cell	-
2-Acetamidofluorene	AAF	300	28	5	5	peroral	0.5 w/v% methylcellulose	negative	Periportal	-	Bile duct, Oval cell	-
N-Nitrosodiethylamine	DEN	30	28	5	5	peroral	0.5 w/v% methylcellulose	negative	-	Centrilobular	Bile duct	-
Ethambutol	ETB	1000	28	5	5	peroral	0.5 w/v% methylcellulose 0.5 w/v%	negative	-	Single cell	-	Centrilobular
Carbon tetrachloride	CCL4	100	3	5	5	peroral	corn oil	negative	Centrilobular	-	-	-
Lomustine	LS	6	3	5	5	peroral	0.5 w/v% methylcellulose 0.5 w/v%	negative	-	-	-	-

### Lipid extraction

Plasma (20 μL) was mixed for 1 min with methanol (180 μL) containing 2 μM phosphatidylethanolamine (PE; 12:0/12:0; Avanti Polar Lipids, Alabaster, AL) as an internal standard. After mixing, the homogenate was centrifuged at 15,000 *g* for 4 min to precipitate the cellular debris. The supernatant was collected, filtered, and stored at a temperature of − 80 °C until use.

### Lipid measurements

Measurements of the lipid content were performed as per the following protocol. The samples were randomized for the LC/MS measurement. Lipid extracts were separated by the LC system using an InertSustain Bio C18 column (2.1 × 150 mm, 1.9 μm; GL Sciences, Tokyo, Japan) connected to an Ultimate 3000 HPLC system (Thermo Fisher Scientific, Waltham, MA). A binary solvent system was used, in which the mobile phase A consisted of acetonitrile:methanol:H_2_O (9:9:2) with 10 mM ammonium formate, and 0.1 % formic acid, and the mobile phase B consisted of isopropanol: acetonitrile (9:1) with 10 mM ammonium formate and 0.1 % formic acid. The flow rate was set at 200 μL/min and the column oven was held at 55 °C. Before gradient elution, the column was equilibrated with 0 % mobile phase B. Samples (5 μL) were injected, and the gradient elution was initiated at 0 % mobile phase B for 0.1 min. It was then increased to 40 % mobile phase B from 0.1 to 5 min; and then further increased to 55 % mobile phase B from 5 to 10 min. The gradient elution was then finally increased to 95 % mobile phase B from 10 to 15 min, before maintaining it at 95 % mobile phase B for a further 10 min. The column was equilibrated with 0 % mobile phase B for 15 min before the next sample was injected. Subsequently, the lipid compositions were determined and measured by the MS system using a Q Exactive mass spectrometer (Thermo Fisher Scientific). The spray voltage was set to 3.5 and − 2.5 kV in positive and negative ion modes, respectively. The following conditions were held constant for both the positive and negative ion modes. MS detection was performed with alternation between the high mass accuracy full scan (full MS) and data-dependent MS2 scan (dd-MS2) in both the positive and negative ion modes; dd-MS2 was set to Top 5. The scan resolutions for full MS and dd-MS2 were 35 000 and 17 500, respectively. The scan range of the MS was set to m/z 300–1600. Representative chromatograms of the plasma obtained from the rats treated with CCl_4_ and LS for 28 days are presented in Additional file 1: Figure S1.

### Data processing

For the lipid quantification, the raw full MS data obtained by LC/MS were processed using the 2DICAL software (Mitsui Knowledge Industry, Tokyo, Japan), which allowed the alignment of the detected ion peaks of each biomolecule obtained at a specific m/z with the column retention time (RT). The main parameters of the 2DICAL software were set as described previously, with a few modifications [20]. The absolute intensity threshold of the ion detection was set to 50 000 and 500 000 for the peak extraction in the negative and positive ion modes, respectively. For the alignment, the RT, m/z, and detection tolerances were set as 0.25, 0.1 Da, and 20 %, respectively. For samples with missing values for a lipid, 50 000 (negative ion mode) or 500 000 (positive ion mode) was applied. The intensities of each extracted ion peak were normalized to those of the internal standard (PE [12:0/12:0]). Subsequently, the lipids in the extracted ion peaks were identified via the processing of the raw full MS and dd-MS2 data using the Lipid Search software (Mitsui Knowledge Industry, Tokyo, Japan) in the product search mode. This allowed the attribution of the lipids’ identities from a combination of an accurate m/z of the precursor ions and dd-MS2 spectral patterns. The precursor and product tolerance were set to 10 and 15 ppm, respectively. All identified lipids are listed in Table 2 and Additional file 2: Table S1. If more than two lipid species possessed the same chemical formula, alphabetical letters were added after each lipid class to distinguish them from each other. All the obtained data were normalized to the average of each control (the adjusted average control value was set as 1). The normalized data, fold changes, and associated *p*-values of all the samples from the experimental groups, when compared with the controls, are listed in Additional file 2: Table S2 and Additional file 2: Table S3. The molecules with the same formula but different fatty acid compositions assessed by their retention times were discriminated by small alphabetical letters, a and b, at the end of their names.

**Table 2:** Identified lipid classes and the numbers of individual lipid molecules

Lipid type	Lipid classes	Number of molecules
Phosphoglycerolipid	Lysophosphatidylcholine (LPC)	15
	Lysophosphatidylethanolamine (LPE)	6
	Phosphatidylcholine (PC)	43
	Ether-type PC (ePC)	6
	Phosphatidylethanolamine (PE)	10
	Ether-type PE (ePE)	8
	Phosphatidylinositol (PI)	9
Sphingolipid	Sphingomyelin (SM)	15
	Ceramide (Cer)	7
	Glycosylceramide (GCer)	1
Neutral lipid	Diacylglycerol (DG)	6
	Cholesterol ester (ChE)	8
	Triacylglycerol (TG)	94
	Total	228

### Principal component analysis (PCA) and statistical tests

For the PCA, the control and drug-treated data sets obtained from the rat plasma were loaded into SIMCA-P+ 12 (Umetrics, Umea, Sweden) and analyzed using PCA-X. To compare the relative plasma levels of the lipids, Welch’s *t*-test was used to assess the statistical difference in plasma lipid levels between the control and drug-treated rats. Differences with *p*-values <0.05 were considered statistically significant.

## Results

### Histopathologic characteristics of the rat livers

Rats treated for 28 days with CCl_4_ exhibited hepatic fibrosis and cellular infiltration in the centrilobular area (Table 1). In contrast, LS treatment for 28 days induced hepatic fibrosis in the bile duct, cellular infiltration in the periportal area, necrosis in the midlobular area, and the proliferation of bile duct epithelial cells and Kupffer cells. The histologic sections of the rat livers treated for 28 days with CCl_4_ and LS are available in the Open TG-GATEs database (http://toxico.nibiohn.go.jp/) [19], and the representative sections are shown in Additional file 1: Figure S2. Rats that were treated with the non-fibrogenic hepatotoxicants, AAF, DEN, and ETB, exhibited no evident hepatic fibrosis by the end of the 28-day treatment protocol. AAF induced cellular infiltration in the periportal area, along with the proliferation of bile duct epithelial cells and oval cells. DEN induced necrosis in the centrilobular area, and proliferation of the bile duct epithelial cells. ETB induced single cell necrosis and a generalized ground-glass hepatocyte appearance in the centrilobular area. Following 3 days of exposure (constituting the pre-fibrosis stage), neither CCl_4_ nor LS exhibited evidence of fibrosis. At that time, CCl_4_ induced cellular infiltration in the centrilobular area; however, rats treated with LS demonstrated no evidence of hepatopathy.

### Plasma lipid profiling of rats treated with CCl_4_ or LS

**Fig. 1:**
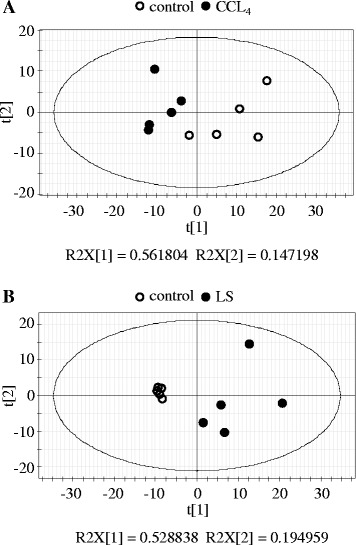
Principle component analysis model of plasma lipid profiles. Plasma lipid profiling data obtained from rats treated with CCl_4_ (**a**) and LS (**b**) for 28 days, and those of their controls

From the qualification of the extracted ion peaks, the lipidomic analysis identified 228 lipids, including 97 phospholipids, 23 sphingolipids, and 108 neutral lipids, in the rat plasma (Table 2 and Additional file 2: Table S1). These 228 lipids were subjected to the following analysis. We first compared the plasma lipid profiles of the rats treated with either CCl_4_ (centrilobular fibrosis) or LS (bile duct fibrosis) with that of the control group in order to establish whether they would demonstrate specific differences in their plasma lipid profiles in comparison with the normal animals. As shown in Fig. 1a, the PCA of the control and CCl_4_-treated rats demonstrated a clear separation between these two groups. The comparison between the PCA of the LS-treated rats and their respective control also demonstrated a clear separation (Fig. 1b). These results indicate that each of the plasma lipid profiles of the two fibrogenic groups were significantly altered compared to those of the controls.

**Fig. 2:**
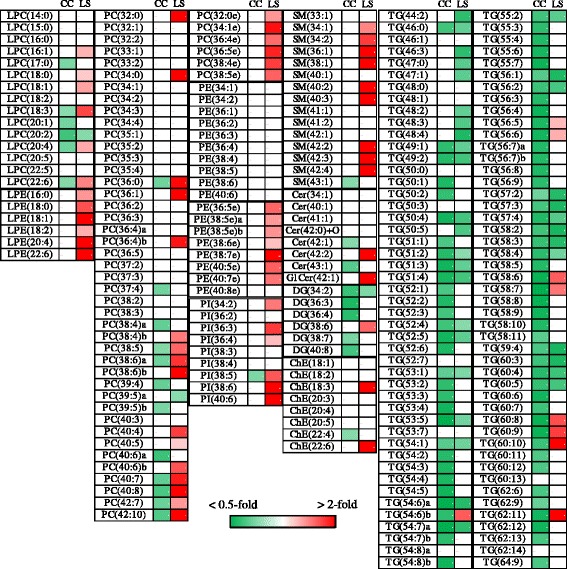
Heat maps of plasma lipid profiles of rats treated with CCl_4_ or LS for 28 days. (*p* < 0.05) lipid molecules was generated using the mean fold changes in the levels of molecules, calculated as ratios against each control. Vacant white cells indicate lipids that did not show significantly different levels. CC; carbon tetrachloride, LS; lomustine. The molecules with the same empirical formula but different structure, as assessed by the retention time, were denoted by the addition of lower-case alphabetical suffixes. Other abbreviations were described in Table 2

We then compared and contrasted the alterations in the plasma lipid profiles caused by CCl_4_ and LS. As shown in Fig. 2, the alterations in the plasma lipid profiles compared with the control group were clearly different between the CCl_4_- and LS-treated groups, with some exceptions. In the case of the rats treated with CCl_4_, there were decreases in the circulating levels of some lysophosphatidylcholines (LPCs), phosphatidylcholines (PCs), diacylglycerols (DGs), and triacylglycerols (TGs). Conversely, in the rats treated with LS, there were increases in the circulating levels of some LPCs, lysophosphatidylethanolamines (LPEs), PCs, ether-type PCs, ether-type PEs, phosphatidylinositols (PIs), sphingomyelins (SMs), ChEs, and highly-unsaturated TGs. Only a few lipids, mainly TGs such as TG(52:4), TG(54:6)a, and TG(56:1), were commonly decreased in this group. Therefore, it is clearly demonstrated that the lipid profiles of the plasma are altered in very different ways by the specific hepatopathies caused by CCl_4_ and LS.

### Specific lipid alterations in rats with CCl_4_- and LS-induced hepatic fibrosis

Having demonstrated that CCl_4_ and LS alter the lipid profile in markedly different ways, we next explored whether these alterations were caused specifically by hepatic fibrosis in their respective regions (centrilobular versus portal), as opposed to non-fibrotic hepatopathies in the same regions.

**Fig. 3:**
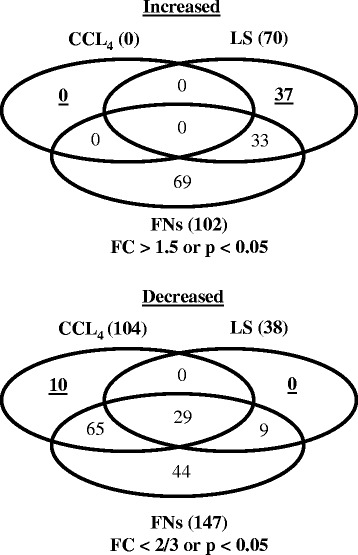
Venn diagrams of the number of lipid molecules that showed significantly different levels between the control and chemical-treated rats (CCl_4_, LS and non-fibrogenic chemicals for 28 days). Increased: chemical-treated rats > control, decreased; chemical-treated rats < control. CCl_4_, carbon tetrachloride; FC, fold change; FNs, non-fibrogenic chemicals; LS, lomustine

To achieve this, we first compared the alterations in the plasma lipid profiles of CCl_4_ and LS with those of three non-fibrogenic hepatotoxic chemicals (AAF, DEN, and ETB) following a 28-day treatment period. Aspects of the plasma lipid profiles altered by any of these non-fibrogenic hepatotoxins by more than 50 % when compared with the control group, or the demonstration of a statistically significant difference (*p* < 0.05) from the control group, were defined as changes that were unrelated to fibrosis. As shown in Fig. 3, of all the lipid levels that decreased following CCl_4_ treatment, only 10 out of 104 were specifically altered by CCl_4_, suggesting that they may be specific changes seen in centrilobular fibrosis. In contrast, of all the lipid levels increased by LS, 37 out of 70 were specifically increased by LS, which suggests that they may be specific changes seen only in bile duct or periportal fibrosis. There was no common specific lipid alterations among CCl_4_ and LS, further indicating that the alterations in their plasma lipid profiles may be specific to the region of the liver unit that undergoes fibrosis.

**Fig. 4:**
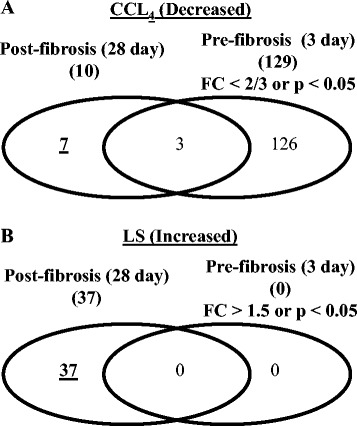
Venn diagrams of the number of CCl_4_- (**a**) or LS- (**b**) specific lipid molecules that showed significantly different levels between the control and chemical-treated rats (CCl_4_ and LS for pre-fibrosis and post-fibrosis stages). CCl_4_; carbon tetrachloride, FC; fold change, LS; lomustine

**Fig. 5:**
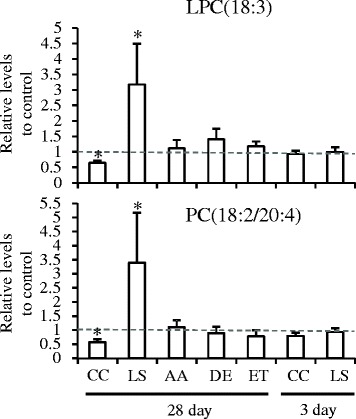
Representative lipid molecules altered only in the fibrosis state. Data are presented as the normalized levels to the control and shown as the mean ± standard deviation. *p* < 0.05 in paired t-tests comparing the control and chemical-treated rats. AA; 2-acetamidofluorene, CC; carbon tetrachloride, DEN; N-nitrosodiethylamine, ETB; ethambutol, LS; lomustine. Other abbreviations are described in Table 2

To further specify the lipid alterations resulting from hepatic fibrosis, we next compared the specific lipid alterations elicited by CCl_4_ or LS with those at the pre-fibrosis stage (3-day treatment), in which neither CCl_4_ nor LS had induced fibrosis. Alterations in the plasma lipid levels at the pre-fibrosis stage of >50 % compared with the control group, or displaying a statistically significant change (*p* < 0.05), were defined as changes that were unrelated to fibrosis. As shown in Fig. 4, of the ten specific lipids altered by CCl_4_, seven were specific to fibrosis (28-day treatment), whilst three were unrelated to fibrosis. These seven lipids included three LPCs, three PCs, and one ceramide (Table 3). In contrast, all of the 37 specific lipids altered by LS treatment were specific for fibrosis (Fig. 4). These included four LPCs, 14 PCs, three ether-type PEs, two PIs, five SMs, and nine TGs (Table 3). Of these lipids, three LPCs (LPC[18:3], LPC[20:4], and LPC[22:6]), and three PCs (PC[18:2/20:4 (38:6a)], PC[40:8], and PC[20:4/22:6 (40:10)]) were listed in both CCl_4_ and LS, although the responses to these chemicals were different. As an example, the changes in the listings of LPC(18:3) and PC(18:2/20:4) are shown in Fig. 5.

**Table 3:** Candidate lipid markers for hepatic fibrosis induced by CCL_4_ and LS

Class	Ion	Fatty side chains	CCL_4_	LS
LPC	LPC(18:0)	18:0	−	↑
**LPC**	**LPC(18:3)**	**18:3**	**↓**	**↑**
**LPC**	**LPC(20:4)**	**20:4**	**↓**	**↑**
**LPC**	**LPC(22:6)**	**22:6**	**↓**	**↑**
PC	PC(34:0)	16:0/18:0	**−**	**↑**
PC	PC(36:0)	18:0/18:0	**−**	**↑**
PC	PC(36:4)b	16:0/20:4	−	↑
PC	PC(38:4)b	18:0/20:4	−	↑
PC	PC(38:5)	18:1/20:4	−	↑
**PC**	**PC(38:6)a**	**18:2/20:4**	**↓**	**↑**
PC	PC(38:6)b	16:0/22:6	−	↑
PC	PC(40:4)	18:0/22:4	−	↑
PC	PC(40:5)	20:1/20:4	−	↑
PC	PC(40:6)b	18:0/22:6	−	↑
PC	PC(40:7)	18:1/22:6	−	↑
**PC**	**PC(40:8)**	**N.D.**	**↓**	**↑**
PC	PC(42:7)	N.D.	−	↑
**PC**	**PC(42:10)**	**20:4/22:6**	**↓**	**↑**
ePE	PE(38:5e)a	N.D.	−	↑
ePE	PE(38:5e)b	N.D.	−	↑
ePE	PE(40:5e)	N.D.	−	↑
PI	PI(36:4)	16:0/20:4	−	↑
PI	PI(40:6)	18:0/22:6	−	↑
SM	SM(34:1)	N.D.	−	↑
SM	SM(34:2)	N.D.	−	↑
SM	SM(40:2)	N.D.	−	↑
SM	SM(42:2)	N.D.	−	↑
SM	SM(42:4)	N.D.	−	↑
Cer	Cer(42:1)	N.D.	↓	−
TG	TG(54:6)b	16:0/18:2/20:4	−	↑
TG	TG(56:5)	16:0/18:1/22:4	−	↑
TG	TG(56:6)	18:1/18:1/20:4	−	↑
TG	TG(58:6)	18:1/18:1/22:4	−	↑
TG	TG(58:7)	N.D.	−	↑
TG	TG(60:8)	N.D.	−	↑
TG	TG(60:9)	18:1/18:2/24:6, 18:1/20:3/22:5	−	↑
TG	TG(60:10)	N.D.	−	↑
TG	TG(62:11)	N.D.	−	↑

*The underline lipid molecules are altered by both CCl*_4_ *and LS. CCl*_*4*_ carbon tetrachloride; *LS* lomustine; *LPC* lysophosphatidylcholine; *PC* phosphatidylcholine; *ePE* ether-type PC, *PI* phosphatidylinositol; *SM* sphingomyelin; *Cer* ceramide; *TG* triacylglycerol; *N.D*. not determined

## Discussion

In the present study, we used plasma lipid profiling to characterize the alterations in the levels of the circulating lipids associated with two different types of hepatic fibrosis in rat models; centrilobular fibrosis that was induced by CCl_4_ and bile duct fibrosis that was induced by LS. Firstly, both treatment with CCl_4_ and LS clearly resulted in lipid alterations when compared with the control. Secondly, the lipid alterations elicited by CCl_4_ and LS were clearly different. Thirdly, a comparison between the treatment with non-fibrogenic hepatotoxins and the pre-fibrotic state of the animals treated with fibrogenic hepatotoxins revealed specific alterations that were induced by CCl_4_ and LS. Decreases in the circulating plasma levels of three LPCs (LPC[18:3], LPC[20:4], and LPC[22:6]) and three PCs (PC[18:2/20:4], PC[40:8], and PC[20:4/22:6]) were found to be specific responses to fibrogenic CCl_4_ treatment, suggesting that these plasma lipid decreases are associated with centrilobular hepatic fibrosis. On the other hand, fibrogenic LS-specific alterations, including increases in the levels of phospholipids (LPCs, PCs, ether-type PEs, PIs, and SMs) and TGs, suggest that bile-duct fibrosis is associated with the alteration of the plasma levels of these lipid classes.

So far, the mechanism underlying the different plasma lipid profiles as a result of CCl_4_ and LS exposure is unclear. However, it has been reported that the levels of specific PCs, such as PC(18:2/20:4) and PC(40:8), decreased during fibrosis progression in the livers of a mouse model of non-alcoholic steatohepatitis [21]. Taken together with this report, centrilobular hepatic fibrosis could induce a decrease in specific PCs, such as PC(18:2/20:4) and PC(40:8), in the liver and then, subsequently, in the circulating plasma. On the other hand, it has been reported that phospholipids were secreted into the bile with bile salts, cholesterol, and bile pigments [22]. In addition, artificial bile duct failure as a result of bile duct ligation leads to increased levels of serum phospholipids [23]. Thus, the increased levels of plasma phospholipids, such as PCs and SMs, as a result of the LS treatment may be associated with the functional failure of the bile secretion of phospholipids via the hastening of bile duct fibrosis. Alternatively, it has been reported that the periportal area of the liver plays a dominant role in fatty-acid degradation [17]. Thus, the increased levels of the plasma lipids as a result of LS treatment may also reflect the loss of periportal liver function for the purpose of lipid catabolism. Taken together, as the zonal heterogeneity of lipid metabolism in the liver has been reported [17, 18], the mechanism underlying the different plasma lipid profiles caused by CCl_4_ and LS exposure might be due to the zone specificity of the area of hepatic fibrosis.

Chronic CCl_4_ exposure also decreased the plasma levels of a broad spectrum of DGs and TGs; however, these were similar to those caused by hepatopathy associated with either chronic exposure to ETB, which did not lead to fibrotic changes (Additional file 1: Figure S3), or with acute CCl_4_ exposure, prior to the onset of the fibrotic changes (Additional file 1: Figure S4). Chronic ETB exposure induced the ground-glass appearance of the hepatocytes of the centrilobular region upon histopathological examination, whilst both the acute and chronic exposure to CCl_4_ induced cellular infiltration into the same area, suggesting that these pathophysiological changes in the centrilobular area by ETB and CCl_4_ could decrease the levels of plasma DGs and TGs. It has been well-documented that the centrilobular area plays a dominant role in lipid synthesis, including fatty-acid synthesis and esterification [17]. Thus, the deceased levels of plasma DGs and TGs may reflect a loss of the centrilobular liver function.

On the other hand, LS treatment also increased the levels of ether-type PCs, and this increase was observed in rats treated with AAF and DEN (Additional file 1: Figure S3), which led to the proliferation of bile-duct epithelial cells. These observations suggest that the increased plasma levels of ether-type PCs are associated with the proliferation of the bile-duct epithelium. Although the role of ether-type PCs in bile duct proliferation remains unclear, it has been reported that higher levels of ether-type PCs were detected in migratory breast cancer cells [24]. Thus, this increased level of plasma ether-type PCs might be associated with the migratory process of the bile duct epithelial cells.

In non-alcoholic steatohepatitis, which is one of the major causes of hepatic fibrosis, the levels of plasma lipids such as TGs and cholesterol were elevated and their levels were inversely associated with the resolution of non-alcoholic steatohepatitis [25, 26]. In addition, dietary cholesterol worsens non-alcoholic steatohepatitis [27–29]. In the present study, although our platform did not target cholesterol, the levels of TGs were decreased following CCL_4_ treatment. Thus, the elevation in plasma TGs is associated with hepatic steatosis rather than hepatic fibrosis. Conversely, decreased TG and cholesterol levels are associated with liver dysfunction and the progression of liver disease, but not cardiovascular risk, in patients with non-alcoholic, alcoholic, or virus-induced cirrhosis [30–34]. Thus, decreased TG levels might be common features of hepatic fibrosis/cirrhosis and, therefore, are relevant in our CCl_4_-treated hepatic fibrosis model based on plasma lipid profiling. However, the decreased levels of many TGs were observed as a result of the pre-fibrogenic CCl_4_ treatment and non-fibrogenic ETB treatment. Therefore, the precise clinical relevance of our CCl_4_ hepatic fibrosis model remains to be investigated in future studies. On the other hand, hyperlipidemia is an almost universal feature in primary biliary cirrhosis [31]. In the present study, the LS treatment increased the levels of plasma phospholipids, which were related to the artificial bile duct failure as a result of bile duct ligation [23]. Thus, hyperlipidemia might be a common feature of bile duct fibrosis/cirrhosis and, therefore, relevant in our LS-treated bile duct fibrosis model based on plasma lipid profiling.

Several blood markers for hepatic fibrosis have been proposed (i.e. megamitochondria [35], type IV collagen [36], hyaluronic acid [37], dehydroepiandrosterone [38], and Mac-2-binding protein [39]). In the present study, no specific plasma lipid was shared in rats treated with CCl_4_ and LS; the inference being that there is no plasma lipid commonly found in both types of hepatic fibrosis that is affected in the same way. Therefore, we could not consider a plasma lipid as a candidate for a common biomarker of hepatic fibrosis. However, whilst three LPCs and three PCs, such as LPC(18:3) and PC(18:2/20:4), were specifically altered by both CCl_4_ and LS treatments, the responses to these were the opposite of each other. Thus, these lipid molecules are potential markers that can discriminate between different types of hepatic fibrosis.

## Conclusion

In conclusion, we characterized the plasma lipid profiles of rats chronically exposed to two hepatic fibrogenic and three non-fibrogenic hepatotoxins using a lipidomics approach. Chronic CCl_4_ and LS exposure are known to cause centrilobular and bile duct fibrosis, respectively. By comparing the exposure of rats to both agents with a corresponding vehicle control, we revealed that both treatments induced specific alterations in the plasma lipid profiles. In addition, by comparing CCl_4_ and LS with non-fibrogenic hepatotoxicants, as well as the pre-fibrotic stages of CCl_4_ and LS hepatopathy, we demonstrated specific plasma lipid alterations as a result of chronic CCl_4_ and LS exposure that may be indicative of fibrosis in specific regions of the liver lobules. Our study provides an insight into the alteration of the plasma lipidomic profiles, which probably reflect chemical-induced hepatic fibrosis. These findings may help to understand the pathophysiology of different types of hepatic fibrosis.

## Additional files

Additional file 1: Supplementary figures. **Figure S1. ** The representative chromatograms of plasma obtained from rats treated with CCl_4_ and LS for 28 days. **Figure S2.** The representative histologic sections of the livers obtained from rats treated with CCl_4_ (left) and LS (right) for 28 days. **Figure S3.** Heat maps of the plasma lipid profiles of rats treated with CCl_4_, LS, AAF, DEN, and ETB for 28 days. **Figure S4. **Heat maps of the plasma lipid profiles of rats treated with CCl_4_ and LS for 3 (pre-fibrotic stage) and 28 (post-fibrotic stage) days. (PPTX 626 kb) Additional file 2: Supplementary tables. **Table S1. ** Class, m/z, retention time, ion of detected and identified lipid molecules, and determined their fatty side chains. **Table S2.** Normalized levels of lipid molecules in individual samples. **Table S3.** Fold change and p-value of the levels of lipid molecules. (XLSX 317 kb)

## Abbreviations

AAF: 2-acetamidofluorene
CCl_4_: carbon tetrachloride
ChE: cholesterol ester, dd-MS2, data-dependent MS2 scan
DEN: N-nitrosodiethylamine
DG: diacylglycerol
ETB: ethambutol
full MS: high mass accuracy full scan
LC/MS: liquid chromatography and mass spectrometry
LPC: lysophosphatidylcholine
LPE: lysophosphatidylethanolamine
LS: lomustine
PC: phosphatidylcholine
PCA: principal component analysis
PE: phosphatidylethanolamine
PI: phosphatidylinositol
RT: retention time
SM: sphingomyelin
TG: triacylglycerol
