# Supplementary material for: Plasma lipid profiling of different types of hepatic fibrosis induced by carbon tetrachloride and lomustine in rats
Source: Lipids Health Dis. 2016 Apr 12;15:74. doi: 10.1186/s12944-016-0244-1 (PMC4828842; doi:10.1186/s12944-016-0244-1)
Supplement: Additional file 1: — Supplementary figures. Figure S1. The representative chromatograms of plasma obtained from rats treated with CCl4 and LS for 28 days. Figure S2. The representative histologic sections of the livers obtained from rats treated with CCl4 (left) and LS (right) for 28 days. Figure S3. Heat maps of the plasma lipid profiles of rats treated with CCl4, LS, AAF, DEN, and ETB for 28 days. Figure S4. Heat maps of the plasma lipid profiles of rats treated with CCl4 and LS for 3 (pre-fibrotic stage) and 28 (post-fibrotic stage) days. (PPTX 626 kb) [file 12944_2016_244_MOESM1_ESM.pptx]

## Slide 1
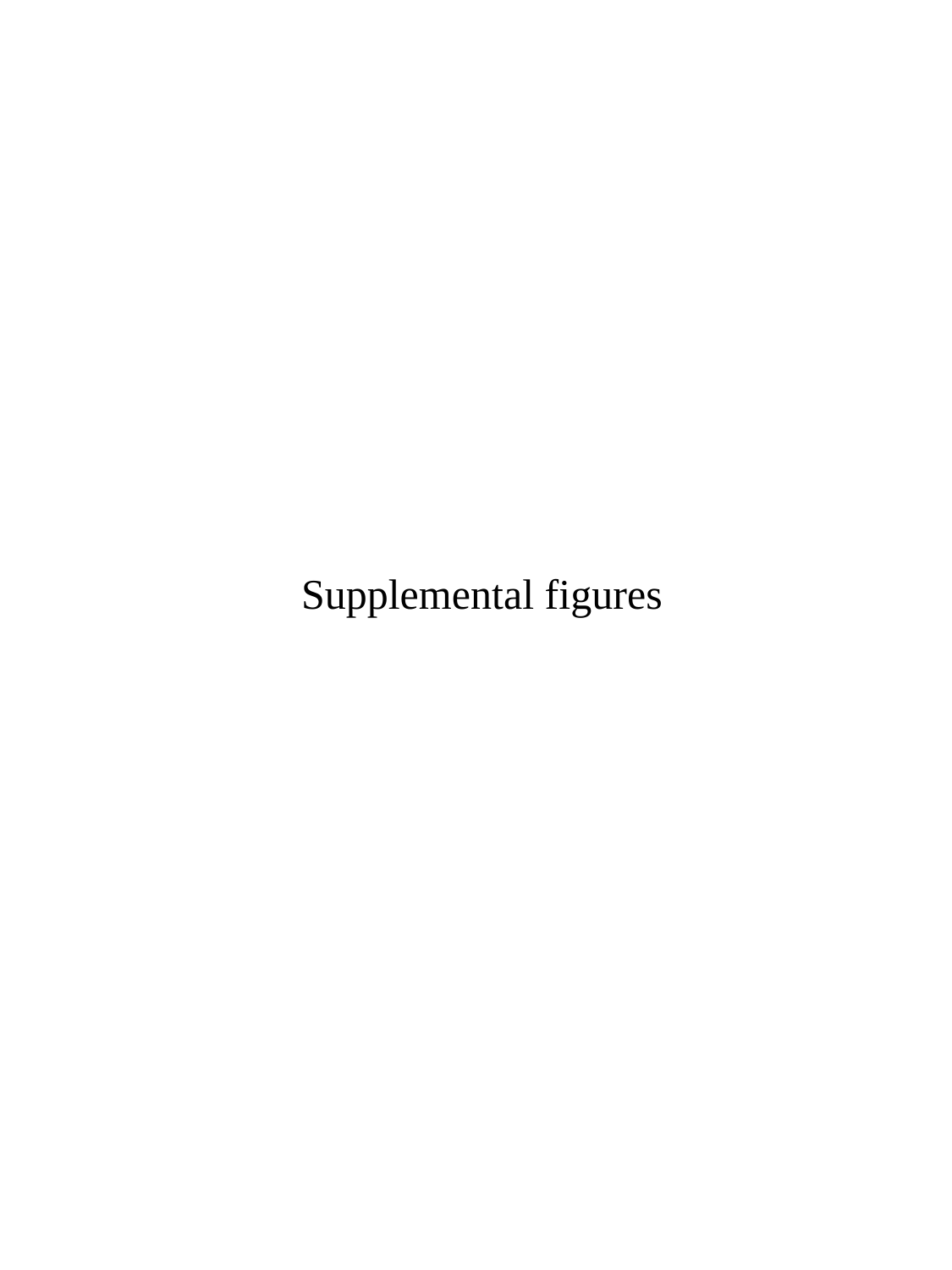

Supplemental figures

## Slide 2
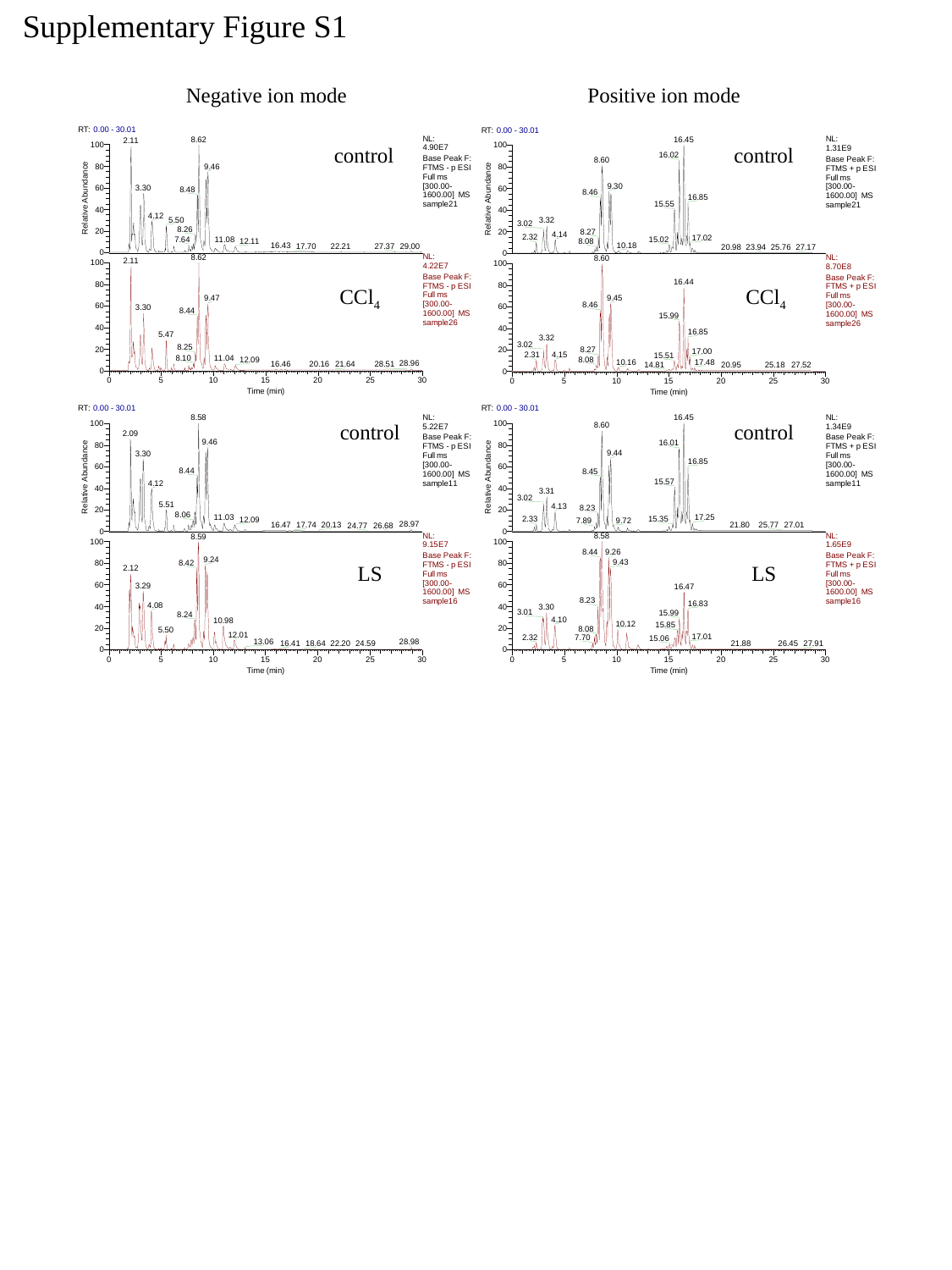

Supplementary Figure S1
Negative ion mode
Positive ion mode
control
control
CCl4
CCl4
control
control
LS
LS

## Slide 3
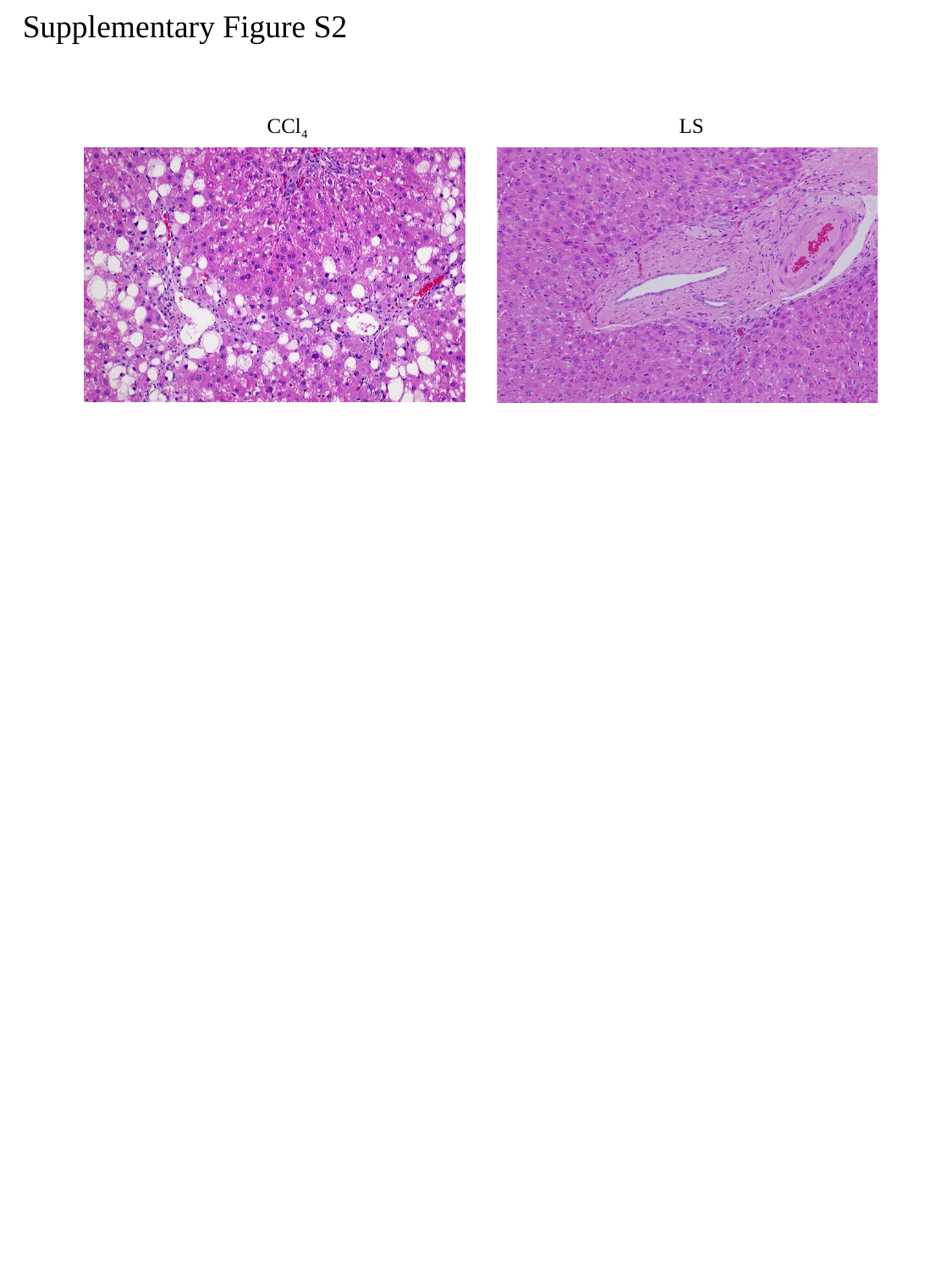

Supplementary Figure S2
LS
CCl4

## Slide 4
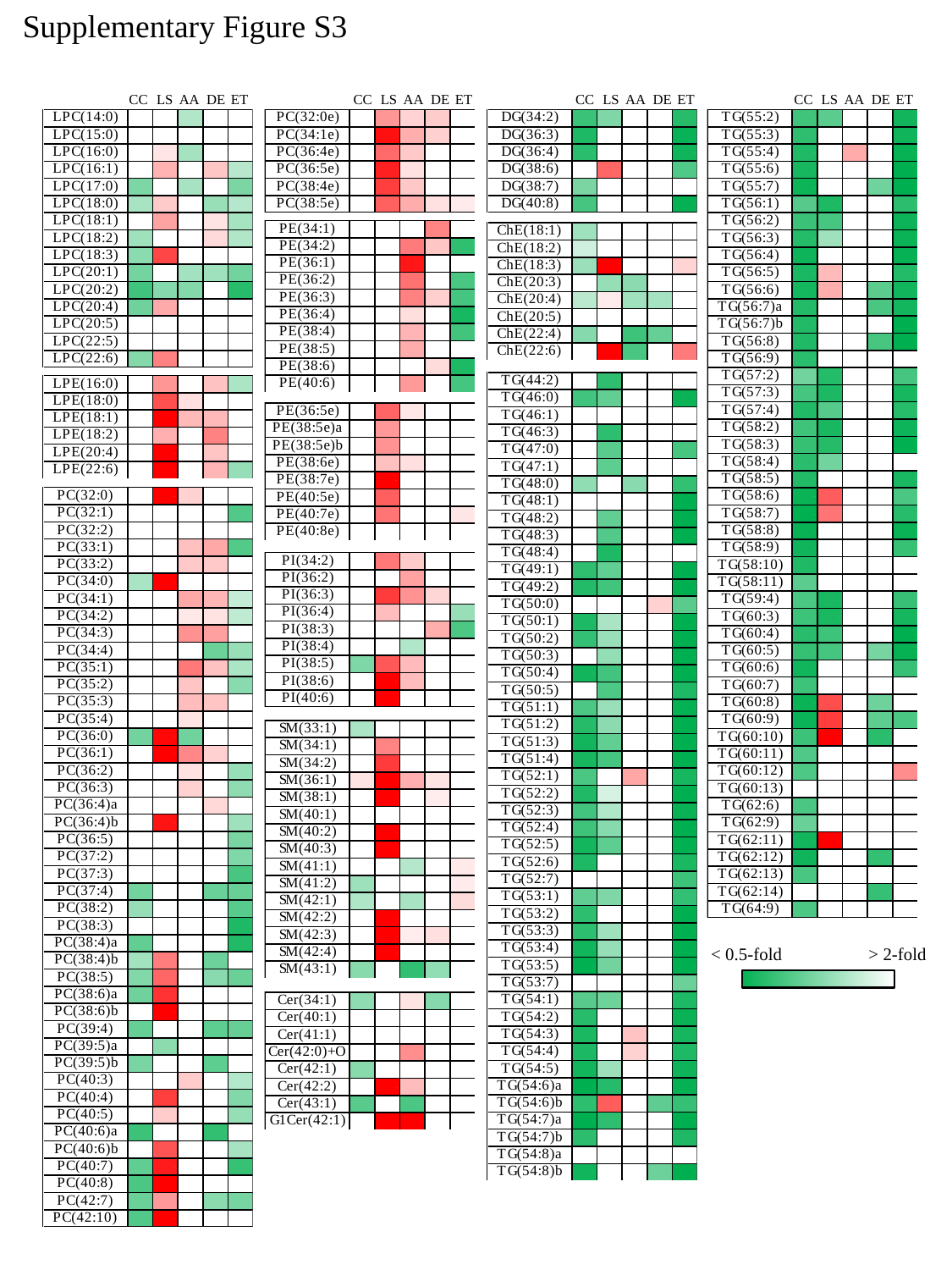

Supplementary Figure S3
CC
LS
AA
DE
ET
CC
LS
AA
DE
ET
CC
LS
AA
DE
ET
CC
LS
AA
DE
ET
> 2-fold
< 0.5-fold

## Slide 5
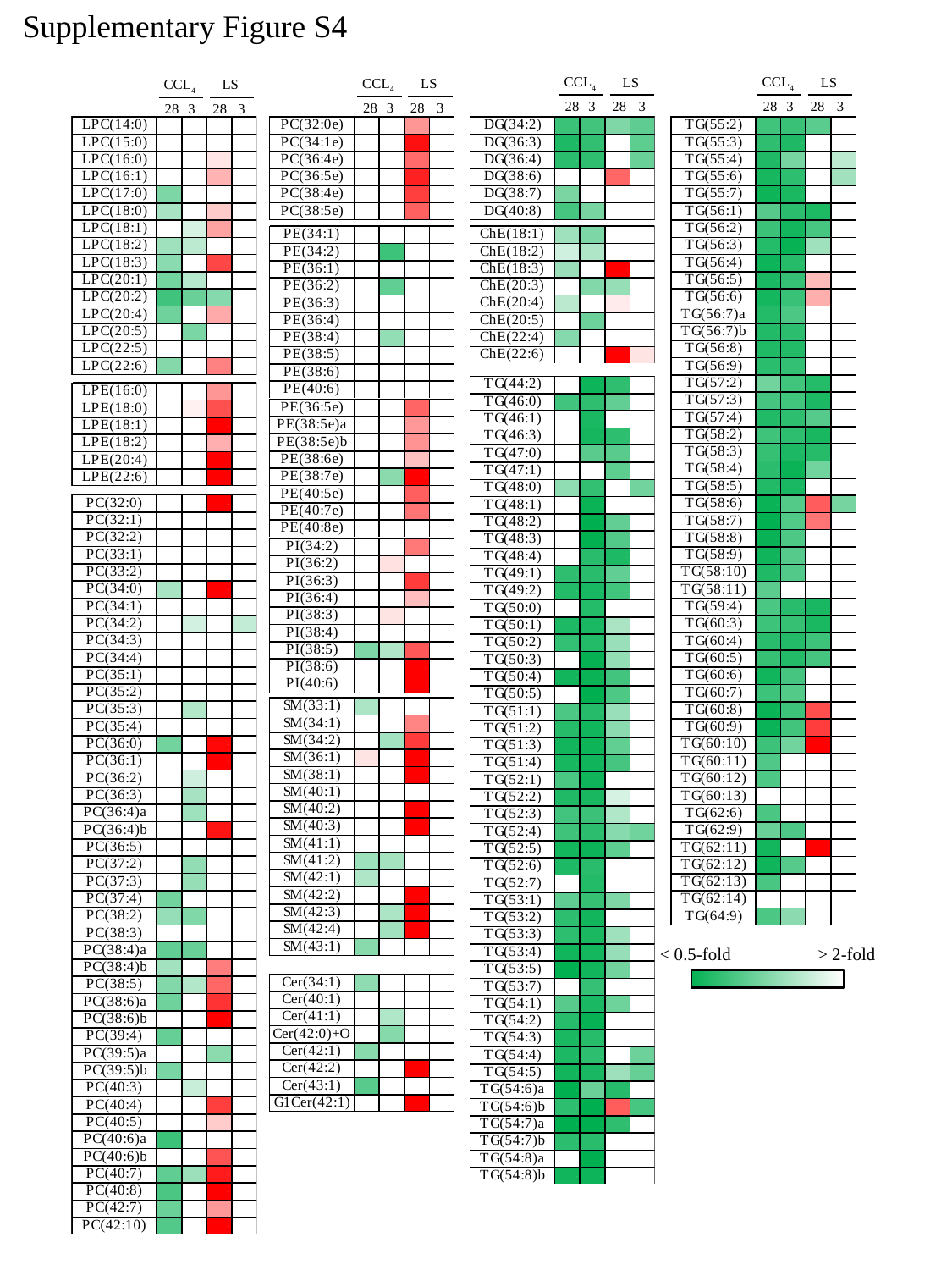

Supplementary Figure S4
CCL4
CCL4
LS
LS
CCL4
LS
CCL4
LS
28
3
28
3
28
3
28
3
28
3
28
3
28
3
28
3
> 2-fold
< 0.5-fold
